# Finding commonalities in rare diseases through the undiagnosed diseases network

**DOI:** 10.1093/jamia/ocab050

**Published:** 2021-05-03

**Authors:** Josephine Yates, Alba Gutiérrez-Sacristán, Vianney Jouhet, Kimberly LeBlanc, Cecilia Esteves, Undiagnosed Diseases Network, Thomas N DeSain, Nick Benik, Jason Stedman, Nathan Palmer, Guillaume Mellon, Isaac Kohane, Paul Avillach

**Affiliations:** Department of Biomedical Informatics, Harvard Medical School, Boston, Massachusetts, USA

**Keywords:** rare diseases, undiagnosed diseases, cluster analysis, supervised machine learning, unsupervised machine learning

## Abstract

**Objective:**

When studying any specific rare disease, heterogeneity and scarcity of affected individuals has historically hindered investigators from discerning on what to focus to understand and diagnose a disease. New nongenomic methodologies must be developed that identify similarities in seemingly dissimilar conditions.

**Materials and Methods:**

This observational study analyzes 1042 patients from the Undiagnosed Diseases Network (2015-2019), a multicenter, nationwide research study using phenotypic data annotated by specialized staff using Human Phenotype Ontology terms. We used Louvain community detection to cluster patients linked by Jaccard pairwise similarity and 2 support vector classifier to assign new cases. We further validated the clusters’ most representative comorbidities using a national claims database (67 million patients).

**Results:**

Patients were divided into 2 groups: those with symptom onset before 18 years of age (n = 810) and at 18 years of age or older (n = 232) (average symptom onset age: 10 [interquartile range, 0-14] years). For 810 pediatric patients, we identified 4 statistically significant clusters. Two clusters were characterized by growth disorders, and developmental delay enriched for hypotonia presented a higher likelihood of diagnosis. Support vector classifier showed 0.89 balanced accuracy (0.83 for Human Phenotype Ontology terms only) on test data.

**Discussions:**

To set the framework for future discovery, we chose as our endpoint the successful grouping of patients by phenotypic similarity and provide a classification tool to assign new patients to those clusters.

**Conclusion:**

This study shows that despite the scarcity and heterogeneity of patients, we can still find commonalities that can potentially be harnessed to uncover new insights and targets for therapy.

## INTRODUCTION

To date, investigators have identified more than 10 000 rare diseases,^1^ most caused by genetic factors.^2^ Several rare diseases do not show obvious symptoms at birth when such presentations often trigger consideration of genetic etiologies.^2^ Thus, they are often un- or misdiagnosed for long periods, leading to delayed treatment and generally worse prognosis.^3^^,^^4^ For example, mucopolysaccharidosis type I non-Hurler phenotype is a lysosomal storage disorder with an estimated prevalence of 1:100 000. Because mucopolysaccharidosis type I non-Hurler phenotype can affect many different organs and tissues to varying degrees, the diagnostic odyssey is protracted with the average age of diagnosis of 28 months.^5^ These delayed diagnostic conclusions lead to unnecessary parental anxiety, patient suffering, complications, inappropriate treatments, and worsened prognosis. The Undiagnosed Diseases Network (UDN) was launched in 2014^6–10^ with the dual aim of reducing diagnostic delays and improving understanding of rare diseases. Participants are evaluated in clinical sites across the United States coordinated by the Coordinating Center. As of May 2019, a total of 1042 patients had been evaluated. As a measure of the UDN’s impact, at the time of the analysis 239 (23%) of those previously undiagnosed patients had now been diagnosed. 

Of the many remaining challenges, we seek how to manage the implicit scarcity and disproportionate heterogeneity of patients with each rare disease to better define a clear diagnosis and understanding of mechanism.^11^ Perhaps the greatest success in rare disease diagnosis has come from tackling each rare disease, one at time accelerated by the development of next-generation sequencing ^12^^,^^13^ technologies and analytic pipelines. The emergence of large population databases of exome and genome sequence data^14^^,^^15^ has contributed to rare disease diagnosis^8^^,^^12^ by providing robust estimates of just how rare (or not) the variants found in these undiagnosed patients are. However, the diagnostic yield of exome sequencing is estimated around 25% to 30% in rare genetic diseases cohorts,^16^^,^^17^ thus leaving a number of patients without a diagnosis. Additionally, genomic sequencing remains expensive, making it less widely available in developing countries with limited resources.^18^ Here, by contrast, we focus on the clinical presentation to leverage the patients’ phenotypic similarity for insight, thus creating a complementary approach. This is challenging because the UDN represents a relatively large patient population of the undiagnosed but in absolute terms is at least 1 or 2 orders of magnitude smaller than studies characterizing common diseases. This analysis of the UDN compares solved and unsolved cases in both pediatric and adult populations. We also present the resulting groups of patients through meaningful comorbidities and associated diagnoses demonstrating the effectiveness and utility of our method. Finally, we provide a classification tool to assign new cases to precomputed clusters, demonstrating how a clinician could leverage the clusters to gain new insights into his patients.

## MATERIALS AND METHODS

We analyzed data from 1042 de-identified UDN patients who were evaluated by clinical experts at the time of the analysis (May 2019). The cohort analyzed included 810 pediatric patients (symptom onset under 18 years of age) and 232 adult patients (onset 18 years of age and older). The data analyzed consisted of general information including age at onset, age at UDN evaluation, primary symptom reported on application^19^ or assigned by clinical experts during record review,^20^ and Human Phenotype Ontology (HPO) terms that were annotated using PhenoTips by clinicians (with no number restriction).^21^ UDN data are available to approved researchers only. We integrated the UDN data into the UDN PIC-SURE platform.^22^ Analysis was performed using Python 3.5.2 leveraging the PIC-SURE^23^ API on the High Performance Data Storage platform. Open-source code is publicly available online (https://github.com/hms-dbmi/UDN-gateway-clusters). The study was approved by the central Institutional Review Board at the National Human Genome Research Institute (registration number 00000014).

### Descriptive analysis

We analyzed the pediatric and adult cohorts by age, race, ethnicity, primary symptoms, and clinical site of evaluation, subcategorizing each as diagnosed or undiagnosed. We observed the breakdown of patients into the 23 top-level phenotypic groups determined by the HPO ontology.^24^ Statistical difference between groups was computed using the Mann-Whitney *U* test.^25^

### Network generation and cluster detection

**Figure 1.**
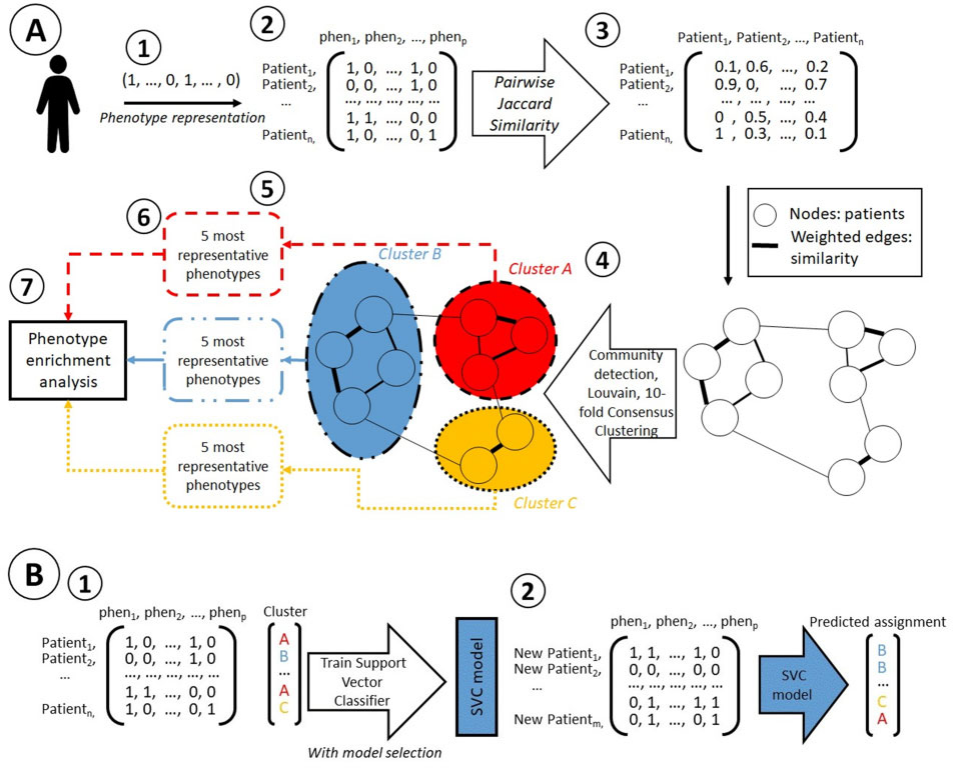
(A) Workflow for the clustering and phenotype enrichment analysis per cluster of the Undiagnosed Diseases Network. (B) Workflow for the training of support vector classifier (SVC) and assignment of new patients. (A) (1) Patients were represented as binary vectors (1 if the phenotype was present, 0 if absent). (2) All patient vectors were aggregated in one matrix representing the whole network. (3) The pairwise Jaccard similarity was computed for every pair of patients and represented a similarity matrix. Similarities ranged from 0 to 1. (4) A network of patients was created, the nodes representing patients of the Undiagnosed Diseases Network, the edges being proportional to the similarity between the 2 nodes that they linked. Patients with a 0 score for similarity were not linked. (5) The Louvain community detection algorithm was performed on the network, and clusters were detected. (6) The list of the 5 phenotypes that were presented by the highest proportion of patients within the cluster was extracted and referred to as the list of best phenotypes. (7) The proportion of patients presenting the list of best phenotypes was represented as a heatmap. (B) (1) An SVC is trained on the patients labeled with their cluster number. (2) New patients represented with their Human Phenotype Ontology annotations are assigned to clusters with the trained SVC.

As illustrated in Figure 1A, the network of UDN patients was created by computing the Jaccard similarity^26^ between patients using explicitly annotated HPO (negative associations were discarded for clustering). The HPO terms used were manually mapped by experts, thus encompassing both leaf terms of the ontology (eg, hypoargininemia) or higher-order terms (eg, seizures). We did not explicitly annotate parent terms of the expert mapped HPO terms. To compute the pairwise Jaccard similarity, we first represented each patient as a binary vector (1 denoting if a term has been positively annotated by an expert, 0 if it was negatively or not annotated). We then computed the Jaccard similarity JS for each pair of patients, where the Jaccard similarity $JS\left(\mathrm{patient}\;1,\;\mathrm{patient}\;2\right)=\frac{\left|\mathrm{patient}\;1\;\cap \;\mathrm{patient}\;2\right|}{\left|\mathrm{patient}\;1\;\cup \;\mathrm{patient}\;2\right|}$. Clusters were identified by the Louvain community detection method,^27^ with 10-fold consensus clustering,^28^ python packages python-louvain^29^ and netneurotools,^30^ using resolution (3.0 for adult and 1.2 for pediatric) as an algorithmic parameter to define the granularity of cluster detection. Patients were assigned labels according to their cluster membership. We performed cluster detection separately for the pediatric population and adult population.

### Cluster analysis and phenotype enrichment analysis

We described the clusters by the following variables: number of patients per cluster; female-to-male ratio; average number of HPO terms per patient; odds ratio of the presence of a diagnosis, given cluster inclusion; age (years) at onset of symptoms; and age (years) at UDN evaluation (see regions 6 and 7 of Figure 1A). For each cluster, we further represented the proportion of patients exhibiting the 5 most representative phenotypes as a heatmap. The significance of distribution differences between all clusters was calculated with the Kruskal-Wallis H test.^25^

### Cluster diagnostic characterization and classification

We described how the known diagnosed conditions are distributed among clusters. We then train 2 support vector classifiers (SVCs) on the UDN dataset, one using pairwise Jaccard similarity and the other using raw HPO binary vectors. The pairwise Jaccard similarity matrix was computed calculating the similarity between the raw HPO representation of test set patients and training set patients for each pair of test/training patients. We trained our models on 90% of the data with 10-fold stratified cross-validation for model selection and tested on the remaining data. We then used the trained SVCs to assign 8 rare diseases with reported HPO annotation—Rett syndrome, Hurler syndrome, facioscapulohumeral muscular dystrophy, familial dysautonomia, Hutchinson-Gilford progeria syndrome, spinocerebellar ataxia, epileptic encephalopathy (EE), and myofibrillar myopathy—to our computed clusters. (see Figure 1B).

### Validation of cluster comorbidities in national claims database

We repeated our method using a national claims database of 67 million patients, containing individuals that filed a claim through a specific provider, thus encompassing patients with a common disease, a rare disease, or even no disease. We mapped the 10 most representative phenotypes—as defined by HPO terms—of each cluster to PheWAS codes (PheCode)^31^ using an expert mapping manual—a document containing correspondences between HPO and PheWAS—created by Bastarache et al.^32^ We then mapped PheCodes to International Classification of Diseases–Ninth Revision (ICD-9) or –Tenth Revision (ICD-10) terms,^33^ using PheWAS catalog, version 2.^31^ There were 63 HPO terms that represented the 10 most representative phenotypes for both adult and pediatric clusters because of overlap. We mapped 950 ICD-10 and 458 ICD-9 terms to 59 HPO terms (of the 63 HPO terms). For each cluster’s UDN phenotypes, we selected the 10 comorbidities that were presented by the highest proportion of patients within the cluster. We then searched the claims database for patients that presented simultaneously these 10 comorbidities (we also performed complementary analysis searching for patients presenting only 9, 8 or 7 of the 10 HPO terms). The inclusion criteria were patients with at least 1 ICD-9/10 code that maps to a HPO term, at least 12 months of coverage with medical information (from January 2008 to December 2019), and comparable age of diagnosis. Comorbidities were defined as any 2 codes occurring for a specific patient during the time of enrollment in the database (without setting any time window). We used bootstrap sampling for *P* value inference, comparing the counts with 1000 randomly selected sets of 10 HPO terms (we found significant co-occurrence of the 10 most representative comorbidities in 3 out of 4 mappable clusters, for both pediatric and adult clusters; results can be found in Supplementary Appendix and Supplementary Figure 4).

## RESULTS

### Descriptive analysis

At the time of this study, 1042 patient records were present in the PIC-SURE UDN database (Table 1). We divided them into pediatric onset of symptoms (78%) and adult onset of symptoms (22%). Nearly a quarter of patients were labeled as “diagnosed.” Of these 239 diagnosed patients, 45 had adult-onset symptoms (19%) and 194 had pediatric-onset symptoms (81%). The female-to-male ratio was 1.04 (532:510). Average age at UDN evaluation was 20 (interquartile range [IQR], 5-31) years. Average age at symptom onset was 10 (IQR, 0-14) years. The diagnosed pediatric population was significantly younger at symptom onset (*P* = .002) and UDN evaluation (*P* = .004) as compared with the undiagnosed pediatric population: 2 (IQR, 0-1) years vs 3 (IQR, 0-3) years for symptom onset and 11 (IQR, 4-14) years versus 12 (IQR, 4-17) years for UDN evaluation. The majority (81%) of the population was White. Primary symptoms (self-reported) were mainly neurological (48%) and musculoskeletal (12%). Patients were almost evenly distributed among the evaluation sites (12% per site).

**Table 1.** Analysis of the UDN database as of May 6, 2019, depicting age, race, ethnicity, primary symptoms, and clinical sites of evaluation

	Attribute	Adult Diagnosed	Adult Undiagnosed	Pediatric Diagnosed	Pediatric Undiagnosed	All	Mann-Whitney *U P* Value
Female-to-male ratio	—	22:23	102:85	113:81	295:321	532:510	(Fisher) Adult: .51
							Pediatric: .013
Age	At symptom onset, y	36 (26-44)	38 (26-50)	2 (0-1)	3 (0-3)	10 (0-14)	Adult: .19
							Pediatric: *P*<.001
	At UDN evaluation, y	45 (33-57)	47 (36-58)	11 (4-14)	12 (4-17)	20 (5-31)	Adult: .24
							Pediatric: <.001
Race	White	36 (16)	156 (67)	149 (18)	498 (61)	839 (81)	/
	Asian	5 (2)	7 (3)	17 (2)	34 (4)	63 (6)	
	American Indian or Alaska Native	0 (0)	0 (0)	0 (0)	3 (<1)	3 (<1)	
	Black or African American	2 (1)	14 *(*6)	11 (1)	21 (3)	48 (5)	
	Native Hawaiian Pacific Islander	0 (0)	0 (0)	0 (0)	2 (<1)	2 (<1)	
	Other	2 (1)	10 (4)	17 (2)	58 (7)	87 (8)	
Ethnicity	Not Hispanic or Latino	33 (14)	152 (66)	132 (16)	452 (56)	769 (74)	/
	Hispanic or Latino	1 (<1)	12 (5)	40 (5)	103 (13)	156 (15)	
	Unknown/not reported ethnicity	11 (5)	23 (10)	22 (3)	61 (8)	117 (11)	
Primary symptom	Neurology	22 (9)	92 (40)	100 (12)	283 (35)	497 (49)	Adult: .08
							Pediatric: .33
	Musculoskeletal	7 (3)	13 (6)	27 (3)	81 (10)	128 (12)	
	Allergies and disorders of the immune system	2 (1)	17 (7)	6 (1)	31 (4)	56 (5)	
	Cardiology and vascular conditions	5 (2)	13 (6)	7 (1)	20 (2)	45 (4)	
	Gastroenterology	0 (0)	2 (1)	6 (1)	29 (4)	37 (4)	
	Rheumatology	2 (1)	10 (4)	0 (0)	22 (3)	34 (3)	
	Endocrinology	0 (0)	4 (2)	6 (1)	12 (1)	22 (2)	
	Pulmonology	0 (0)	4 (2)	4 (<1)	11 (1)	19 (2)	
	Hematology	1 (<1)	4 (2)	1 (<1)	9 (1)	15 (1)	
	Nephrology	2 (1)	4 (2)	3 (<1)	6 (1)	15 (1)	
	Ophthalmology	0 (0)	1 (<1)	1 (<1)	10 (1)	12 (1)	
	Dermatology	3 (1)	3 (1)	2 (<1)	1 (<1)	9 (1)	
	Dentistry and craniofacial	0 (0)	0 (0)	1 (<1)	6 (1)	7 (1)	
	Psychiatry	0 (0)	1 (<1)	2 (<1)	3 (<1)	6 (1)	
	Gynecology and reproductive medicine	0 (0)	0 (0)	1 (<1)	1 (<1)	2 (<1)	
	Infectious diseases	0 (0)	1 (<1)	0 (0)	0 (0)	1 (<1)	
	Urology	0 (0)	0 (0)	0 (0)	1 (<1)	1 (<1)	
	Oncology	0 (0)	1 (<1)	0 (0)	0 (0)	1 (<1)	
	Other	1 (<1)	11 (5)	22 (3)	71 (9)	105 (10)	
Clinical site of evaluation	Baylor	5 (2)	21 (9)	36 (4)	103 (13)	165 (16)	Adult: .5
							Pediatric:.26
	Duke	3 (1)	5 (2)	43 (5)	68 (8)	119 (11)	
	Harvard affiliate	11 (5)	18 (8)	24 (3)	65 (8)	118 (11)	
	NIH	6 (3)	88 (38)	8 (1)	135 (17)	237 (23)	
	Stanford	5 (2)	32 (14)	17 (2)	96 (12)	150 (14)	
	UCLA	3 (1)	12 (5)	38 (5)	69 (9)	122 (12)	
	Vanderbilt	12 (5)	11 (5)	28 (3)	79 (10)	130 (13)	
	WUSTL	0 (0)	0 (0)	0 (0)	1 (<1)	1 (<1)	

Values are n, mean (interquartile range), or n (%). Statistical significance was computed using the Mann-Whitney *U* test (Fisher exact test for female-to-male ratio).

NIH, National Institutes of Health; UCLA, University of California, Los Angeles; UDN: Undiagnosed Diseases Network; WUSTL, Washington University in St Louis.

**Figure 2.**
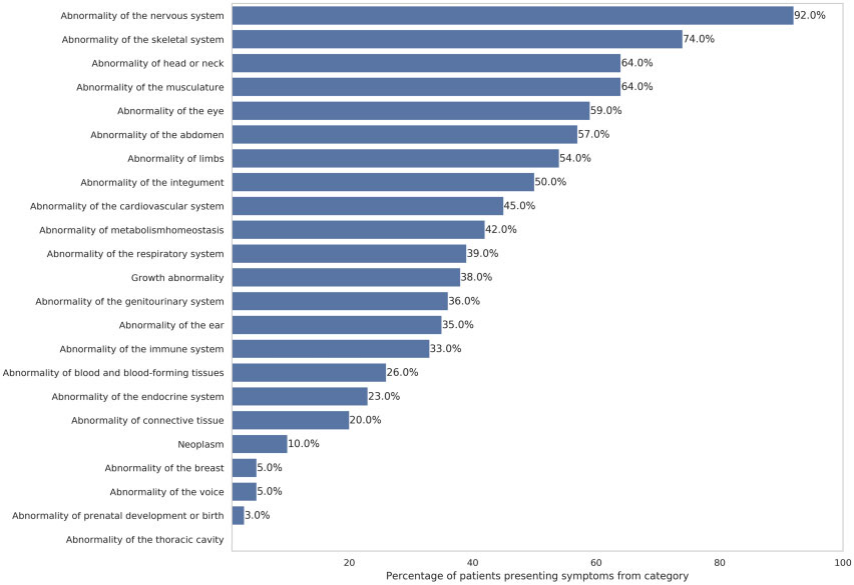
Percentage of patients in Undiagnosed Diseases Network PIC-SURE database presenting at least 1 symptom from top-level phenotypic category in Human Phenotype Ontology. There are 23 types of top-level phenotypic abnormalities in which Human Phenotype Ontology terms can be classified. A single phenotype may be classified within several categories. Each patient was counted within a category if they presented at least 1 symptom classified in the category (as of May 6, 2019).

A total of 3965 unique HPO terms—of 15 247 available terms in the ontology (26%)^34^—were annotated as “positive” or “negative” in the UDN database. The total number of nonunique HPO terms in the UDN database was 26 082 (23 881 positive and 2201 negative), including explicitly annotated parent terms. The average number of HPO terms per patient was 25 (95% confidence interval [CI], 24.0-26.1), including 23 positive (95% CI, 21.9-23.9) and 2 negative (95% CI, 1.8-2.4), with a maximum of 135 terms for a single patient (127 positive and 47 negative) (Supplementary Table 1, Supplementary Figure 1). Patients presented mostly abnormalities of the nervous system (92%), the skeletal system (74%), the head or neck (64%), and the musculature (64%) (Figure 2, Supplementary Table 2, and Supplementary Figure 2).

### Network and clusters

**Figure 3.**
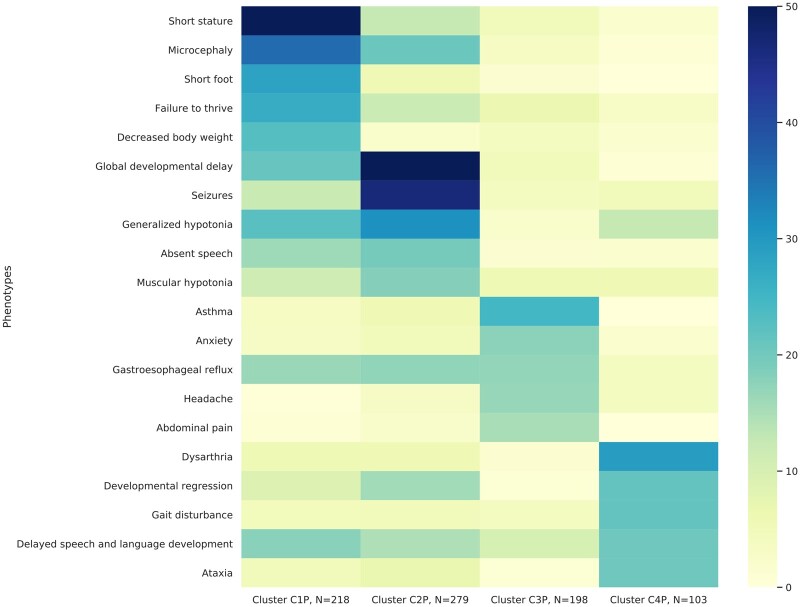
Heatmap of most representative phenotypes for each cluster in the Undiagnosed Diseases Network for networks. The 5 most representative phenotypes for every cluster were extracted. All phenotypes were concatenated in a list referred to as “best phenotypes.” The proportion of patients presenting these phenotypes in every cluster is represented in the heatmap: the darker the shade is, the higher the proportion of patients presenting this cluster-specific phenotype is, ranging from 0% to 50% for adult onset and 0% to 75% for pediatric onset. The cluster sizes are shown next to their name.

We provide the adult analysis in supplementary materials. The pediatric network we constructed was formed by 810 nodes (representing patients) with 157 869 weighted edges (representing the similarity between patients). Louvain community detection identified 4 clusters and 7 outliers (groups of <5 patients) (Table 2 and Figure 3). Outliers were discarded. A total of 798 (99%) pediatric-onset patients were classified within groups of more than 5 patients.

**Table 2.** Analysis of clusters according to the number of included patients, their female-to-male ratio, the average number of HPO terms per patient in the cluster, the odds ratio of being diagnosed, the average age at onset of the disease (years), and the average age at UDN evaluation (years) for pediatric patients (as of May 6, 2019)

	Pediatric				
	Cluster C1P	Cluster C2P	Cluster C3P	Cluster C4P	Kruskal-Wallis *P* Value
Patients per cluster	218	279	198	103	—
Female-to-male ratio	12:10	8:10	10:10	12:10	—
Average of HPO terms per patient	36.1 (33.3-38.9)	20.6 (19.2-22.0)	21.1 (18.9-23.3)	17.9 (16.0-19.7)	<.001
Odds ratio diagnosed (95% CI)	1.7 (1.2-2.4)	1.9 (1.4-2.7)	0.7 (0.4-1.0)	1.4 (0.9-2.2)	—
Average age at onset (95% CI), y	0.7 (0.4-1.0)	0.8 (0.5-1.1)	5.2 (4.4-6.0)	4.8 (3.8-5.8)	<.001
Average age at UDN evaluation (95% CI), y	9.0 (7.7-10.3)	8.4 (7.5-9.3)	18.3 (16.4-20.1)	16.0 (13.5-18.5)	<.001

CI: confidence interval; HPO: Human Phenotype Ontology; UDN: Undiagnosed Diseases Network.

### Cluster phenotype and disease enrichment analysis

Cluster 1 was marked by growth disorders in infancy, specifically short stature (50%), microcephaly (36%), short foot (28%), failure to thrive (27%), and decreased body weight (23%). Notably, this cluster showed a significantly increased probability of diagnosis (odds ratio, 1.7; 95% CI, 1.2-2.2) and was most likely benefit from a UDN-type evaluation. Cluster 2 showed characteristic neurodevelopmental symptoms enriched for hypotonia and microcephaly in infancy, specifically global developmental delay (73%), seizures (47%), generalized hypotonia (31%), microcephaly (21%), and absent speech (19%). Similar to cluster 1, this group bore a significantly increased probability of diagnosis (odds ratio, 1.9; 95% CI, 1.4-2.7) and thus likely benefits from a UDN-type evaluation. Cluster 3 featured a constellation of asthma (24%), gastroesophageal reflux (17%), anxiety (17%), headache (17%), and abdominal pain (16%). Finally, cluster 4 was characterized by neurological symptoms during childhood, specifically dysarthria (29%), developmental regression (21%), gait disturbance (21%), delayed language and speech development (20%), and ataxia (24%).

Of note, each pediatric cluster significantly differed from others by the number of HPO terms per patient (*P* < .001), age at onset of disease (*P* < .001), and age at UDN evaluation (*P* < .001).

### Cluster diagnostic characterization and classification

The pediatric network presented 133 OMIM-annotated^35^ diagnoses (see Supplementary Table 5). Cluster 1 consisted mainly of growth disorders, including neurodevelopmental disorders (5 of the 5 diagnosed in the database), Bainbridges-Ropers syndrome (2 of 2), or Schaaf-Yang syndrome (2 of 2). Cluster 2 exhibited neurological and retardation diseases, such as epilepsy (3 of 5), EE (9 of 10), Rett syndrome (3 of 4), congenital disorder of glycosylation (3 of 4), and hypotonia (2 of 3).

Cluster 3 comprises a set of disorders with more heterogeneous manifestation, such as chronic infantile neurologic cutaneous articular syndrome (1 of 1), Marfan syndrome (1 of 1), and Ehlers-Danlos syndrome (1 of 1). Finally, cluster 4 was characterized by neuromuscular disorders, such as spastic paraplegia (2 of 2), ataxia (2 of 2), and Huntington’s disease (2 of 2). Some diseases were found across several clusters, notably mental retardation (3 in cluster 1, 6 in cluster 2, 1 in cluster 4) or Bethlem myopathy (1 in each of clusters 2, 3, and 4).

The SVC trained on pairwise distances (regularization C = 0.5, balanced class weights, 10-fold cross-validated balanced accuracy: 0.88) reached 0.89 balanced accuracy on the test set (stratified, 80 patients). The SVC trained on the raw HPO binary vector representation (regularization C = 1.9, balanced class weights, 10-fold cross-validated balanced accuracy: 0.82) reached 0.83 balanced accuracy on the test set. Both models classified Hurler syndrome in pediatric cluster 1; EE, Rett syndrome, familial dysautonomia, and facioscapulohumeral muscular dystrophy in cluster 2; myofibrillar myopathy and Hutchinson-Gilford progeria syndrome in cluster 3; and spinocerebellar ataxia in cluster 4.

## DISCUSSION

This report marks the first extensive analysis of the UDN database. It is also the first time in which the data of individuals with many rare diseases have been analyzed as a single population to find hidden links between seemingly disparate conditions. Overall, we discovered pediatric-onset clusters of patients and further determined that these groups were meaningful from a clinical point of view, using both comorbidities and available diagnoses. Finally, we provided a classification tool (SVC) to enable researchers and clinicians to assign previously unseen cases to the clusters we have presented.

The utility of our method is 2-fold. From a clinical perspective, it could be used to complement a practitioner’s observation, by assigning a new patient with annotated HPO terms to a cluster and investigating existing diagnoses in the cluster. The assignment can be performed using the pretrained SVC model (available on github) with any patient with annotated HPO terms, and thus could serve to assign new patients of the same database or utilize another database to uncover new insights. It can also be done using the pairwise distance to training data patients in the UDN database, for authorized investigators. From a research point of view, this grouping could point toward potential shared molecular pathways and justify pooling of samples to answer specific questions: a major drawback of rare diseases studies is the lack of available biopsies or material. Although the clusters presented are of higher order (thus encompassing numerous diseases), this method can be pursued with higher resolution of the Louvain clustering method, enabling an analysis of clusters on a smaller level. Of note, the richness of annotation of patient phenotypes in the UDN database allows clusters to be characterized by symptoms that would not be otherwise considered as primary symptoms.

The rationale behind separating the analysis between adult and pediatric patients was to ensure Louvain detection would not group patients using phenotypes that are strongly correlated with age (eg, create a cluster of arthritic patients).

The main comorbidities found in the clusters had been previously reported as co-occurring and were then confirmed by the clustering of patients with the same diagnoses (in most cases). An overwhelming majority of patients in the database present neurological symptoms, consistent with the prevalence in rare diseases of neurological manifestations.^36^ Cluster 1 can be associated with a number of growth abnormalities syndromes,^37^ with co-occurrence of short stature, small hands or feet, and failure to thrive, or rare disorders^38–40^ with short stature and microcephaly; this was consistent with the prevalence of neurodevelopmental diagnoses in the cluster. Cluster 2 presents developmental delay and epilepsy—often found as comorbidities^41^^,^^42^—as well as hypotonia and additional comorbidities normally seen in several EE.^43^^,^^44^ Once again, the grouping of patients with EE and Rett syndrome was coherent. Cluster 3 is more heterogeneous but can be linked to anxiety disorder symptoms.^45–47^ Cluster 4 presents comorbidities linked to neuromuscular defects consistent with known diseases such as cerebellar ataxia^48–50^ or spastic paraplegia,^51–53^ linked to the found neuromuscular disorders such as ataxia or Huntington’s disease. Some diseases—such as mental retardation—were found across clusters, which could be justified by their heterogeneous manifestations. Overall, cluster 3 is characterized by general symptoms, which can be explained by the heterogeneity of phenotypes presented by the patients within the cluster (with diagnoses such as familial cold autoinflammatory syndrome or factor V deficiency). This cluster might hence benefit from larger granularity to uncover subgroups of diseases.

The assignment of rare diseases represented as “standard patients” confirmed the designation of our clusters: Hurler syndrome was found in the growth disorder cluster, EE and Rett in the neurodevelopmental cluster, SA in the neuromuscular disorder cluster. The assignment of myofibrillar myopathy and Hutchinson-Gilford progeria syndrome points toward the fact that subgroups might be present in cluster 3 (eg, cardiovascular phenotypes); additionally, it corresponds to patients with diseases with very diverse manifestations.

To expose any underlying shared genetic structures between these rare diseases, we developed a computational algorithm to parse for trends in comorbidity that were higher than expected from random chance and found significant co-occurrences. This is supported by the hypothesis that genes disrupted in Mendelian diseases are likely to be disrupted in complex diseases,^54^^,^^55^ thus explaining that comorbidities that co-occur within a rare disease database are likely to co-occur in a national database that is composed of mostly complex traits. Of note, our results were robust when comparing selection of up to 7, 8 or 9 among the best HPO terms and combinations of the randomly selected HPO terms (*P* < .024 for clusters 2, 3, and 4), meaning that even combinations of the most representative terms are overrepresented as compared with chance.

We have successfully unearthed clusters that might be further explored by clinicians or rare disease investigators and families of those who are affected. Our methods can be similarly deployed on other datasets, additionally to methods put forward by other investigators, such as using multiple, repeated measurements of the same patient’s status over time^56–58^ or statistical innovations to improve the accuracy of small sample sizes.^56^^,^^57^

Ultimately, we have shown that despite the scarcity and heterogeneity of patients with diseases that are by definition rare, we can still pinpoint commonalities and potentially harness them to find common pathways or complement a clinicians’ diagnosis.

### Limitations

A preliminary limitation—inherent to the data—is that the UDN database consists mainly of a White population, potentially restricting the application of the described clusters for other ethnicities. This could be circumvented by using a database that does not contain this bias.

A first limitation is that patients in the network were represented using only the presence or absence of phenotype, linked to the information provided. Thus, we could not capture the subtleness of any phenotypic manifestation, such as intensity or frequency of occurrence. In addition, we considered a negative phenotype as equivalent to no information on the presence or absence of a phenotype, thereby discarding it: this can also lead to inaccurate pairing.

The choice of the optimal number of clusters—and thus resolution in this case—remains an open question in unsupervised learning.^59^ However, because the Louvain resolution only determines granularity, changing the resolution would simply break larger clusters into smaller ones, thus not influencing the validity of interpretation.

Of note, the number of diagnosed cases reported is likely underestimated: if a diagnosis is highly likely but uncertain, it will not be flagged as solved in the database.

Finally, our validation studies using the national claims database are subject to several limitations, mainly the scarcity of available correspondence between HPO and PheCodes or ICD-9/10 codes. Indeed, mapping is hindered by the differing objectives of its termino-ontological resources. In this case, HPO centers on phenotypes and PheCodes, ICD-9/10 focuses on diagnoses. At best, this results in inexact matches. At worst, one cannot map at all.^32^ Further work could be pursued by conducting our analysis in a similar manner using similarity with ICD-9/10 code instead of HPO codes.

Although phenotypic clustering might point toward a shared mechanism or etiology, we did not perform an analysis to confirm this hypothesis ourselves: this research would benefit from a subsequent analysis of the genomic data of patients within a cluster. Additionally, our study would also benefit from an external validation group to observe if the assignment of new patients to our clusters is consistent with the associated comorbidities and diagnoses.

## CONCLUSION

We provide a description of the UDN database and a meaningful grouping of its members: patients who exhibit a broad range of rare—and in the majority of cases—undiagnosed diseases. We offer these findings to practitioners and researchers in the rare disease and related fields to explore more deeply, perhaps by integrating genomic or metabolomic data. Such large-scale methods may yield new insights for what is now collectively a large group of patients, who individually cannot garner the necessary resources and attention to their conditions and make significant inroads in early diagnosis.

## FUNDING/SUPPORT

Research reported in this manuscript was supported by the National Institutes of Health Common Fund, through the Office of Strategic Coordination/Office of the National Institutes of Health Director under Award Number U01HG007530. The content is solely the responsibility of the authors and does not necessarily represent the official views of the National Institutes of Health.

## AUTHOR CONTRIBUTIONS

JY conceptualized and designed the study, conducted the analyses, drafted the initial manuscript, and reviewed and revised the manuscript. AG-S conceptualized the study, ran complementary analyses, critically reviewed and revised the manuscript. VJ conceptualized the study and critically reviewed the manuscript. KL and CE coordinated and supervised data collection and critically reviewed the manuscript. TND, NB, and JS provided the computational tools and guidelines to perform the analysis. NP provided additional data and supervised the complementary analyses. GM carried out the initial analyses. IK and PA conceptualized and designed the study, supervised the analysis, and critically reviewed and revised the manuscript. All authors approved the final manuscript as submitted and agree to be accountable for all aspects of the work.

## SUPPLEMENTARY MATERIAL

Supplementary material is available at *Journal of the American Medical Informatics Association* online.

## Supplementary Material

ocab050_Supplementary_Data
