# Supplementary material for: Finding commonalities in rare diseases through the undiagnosed diseases network
Source: J Am Med Inform Assoc. 2021 May 3;28(8):1694–702. doi: 10.1093/jamia/ocab050 (PMC8324228; doi:10.1093/jamia/ocab050)
Supplement: ocab050_Supplementary_Data [file ocab050_supplementary_data.docx]

## **Online-only supplementary materials**

##
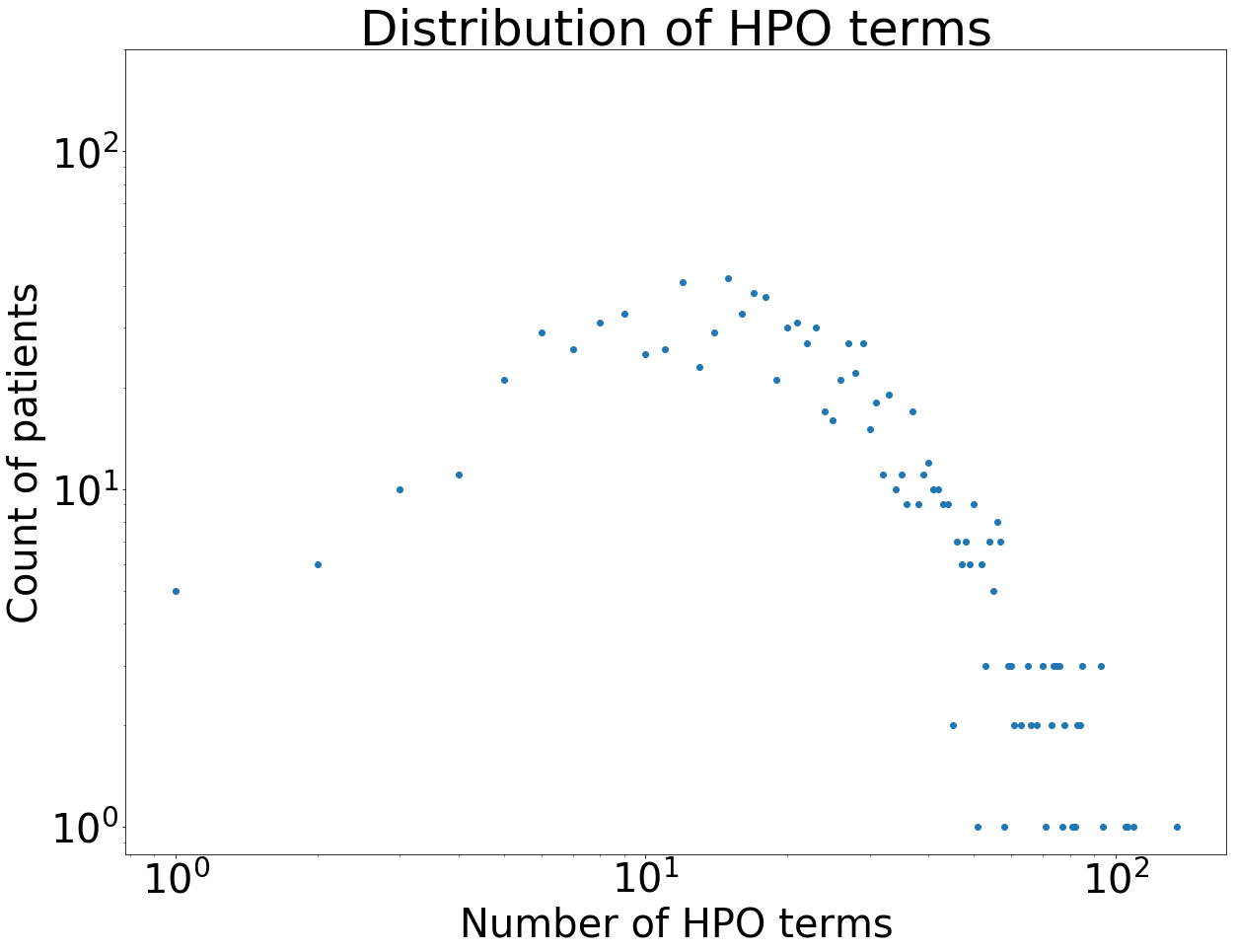


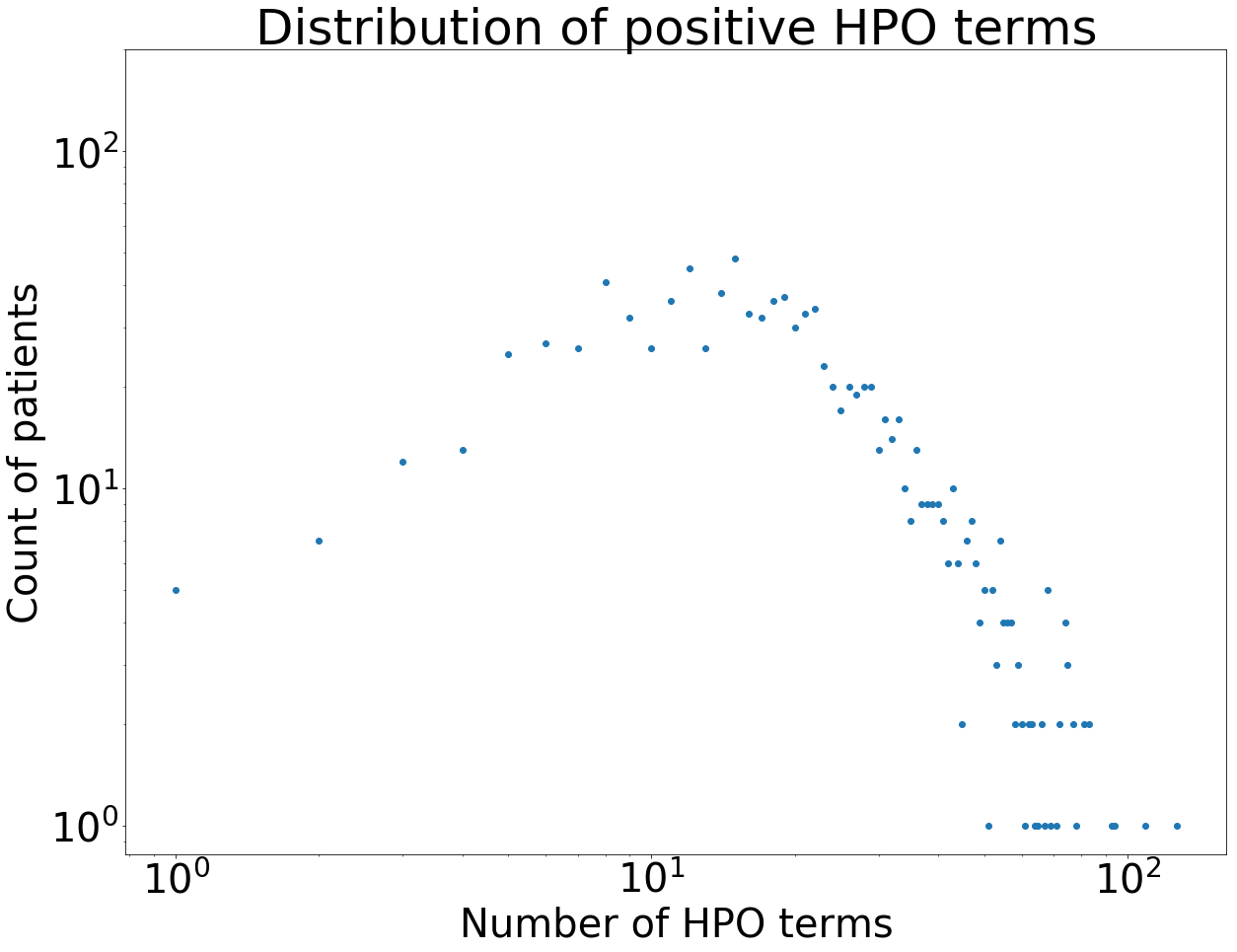

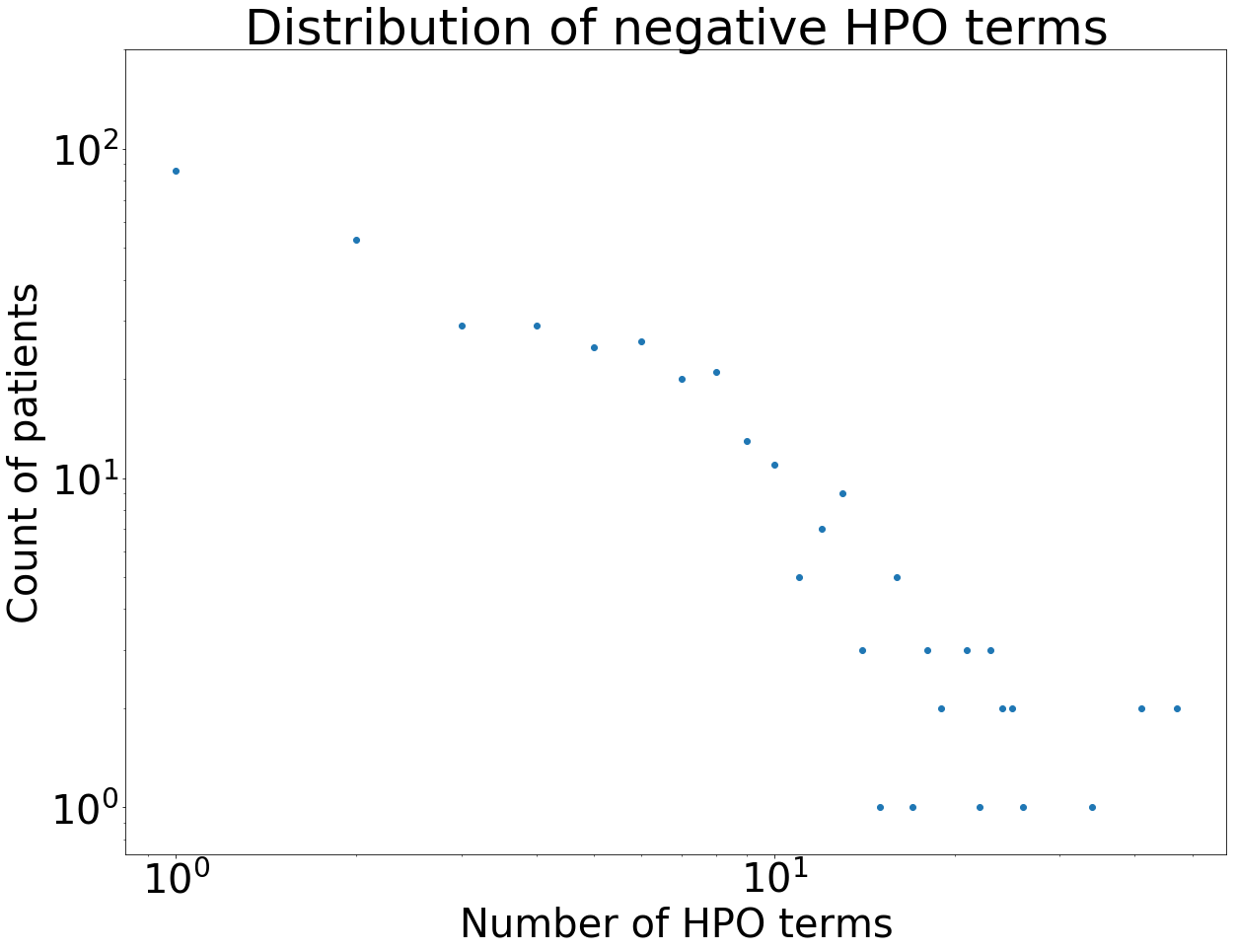


**eFigure 1: Distribution of all positive and negative HPO terms per patient in the UDN database as of May 6, 2019.** The x-axis represents the number of HPO terms per patient (in log-scale). The y-axis represents the number of patients that present with a number of HPO terms (in log-scale).

**eTable 1: Counts of average HPO terms per patient in diagnosed, undiagnosed, and the total population.** The number of HPO terms per patient in the UDN database was computed. The average for those labeled “diagnosed,” “undiagnosed,” and for the total population is shown for positive, negative, and total HPO terms (as of May 6th, 2019).

| HPO terms per patient |  | Adults | | Pediatric | | All |
| --- | --- | --- | --- | --- | --- | --- |
|  |  | Diagnosed | Undiagnosed | Diagnosed | Undiagnosed | / |
| Total | Average | 15.5 | 21.3 | 24.7 | 27.0 | 25.0 |
|  | CI 95% | 12.8 - 18.2 | 19.4 - 23.2 | 22.7 - 26.8 | 25.4 - 28.5 | 24.0 - 26.1 |
| Positive | Average | 14.4 | 18.8 | 23.3 | 24.7 | 22.9 |
|  | CI 95% | 11.9 - 16.9 | 17.1 - 20.6 | 21.4 - 25.2 | 23.2 - 26.1 | 21.9 - 23.9 |
| Negative | Average | 1.1 | 2.5 | 1.4 | 2.3 | 2.1 |
|  | CI 95% | 0.3 - 1.8 | 1.8 - 3.2 | 0.9 - 1.9 | 1.9 - 2.7 | 1.8 - 2.4 |

**eTable 2: Number of positive or negative HPO terms linked to phenotypic abnormalities for groups of diagnosed or undiagnosed patients in the UDN database as of May 6, 2019.** There are 23 types of top-level phenotypic abnormalities in which HPO terms can be classified. A single phenotype may be classified within several categories. For the populations of diagnosed and undiagnosed patients for the pediatric and adult datasets, we show the number of positive or negative phenotypes that are classified within these types.

| Phenotype abnormality | Adult | | Pediatric | | All | |
| --- | --- | --- | --- | --- | --- | --- |
|  | Positive | Negative | Positive | Negative | Positive | Negative |
| Abnormality of blood and blood-forming tissues | 182 | 9 | 383 | 29 | 565 | 38 |
| Abnormality of connective tissue | 54 | 6 | 290 | 31 | 344 | 37 |
| Abnormality of head or neck | 158 | 41 | 3,181 | 131 | 3,339 | 172 |
| Abnormality of limbs | 150 | 6 | 1,642 | 106 | 1,792 | 112 |
| Abnormality of metabolism/homeostasis | 361 | 25 | 717 | 93 | 1,078 | 119 |
| Abnormality of prenatal development or birth | 1 | 0 | 39 | 1 | 40 | 1 |
| Abnormality of abdomen | 259 | 29 | 1,189 | 71 | 1,448 | 100 |
| Abnormality of the breast | 4 | 1 | 61 | 0 | 65 | 1 |
| Abnormality of the cardiovascular system | 302 | 54 | 850 | 143 | 1,152 | 197 |
| Abnormality of the ear | 95 | 22 | 569 | 79 | 664 | 101 |
| Abnormality of the endocrine system | 100 | 4 | 274 | 42 | 374 | 46 |
| Abnormality of the eye | 240 | 80 | 1,403 | 121 | 1,643 | 201 |
| Abnormality of the genitourinary system | 159 | 4 | 515 | 43 | 674 | 47 |
| Abnormality of the immune system | 203 | 20 | 550 | 17 | 753 | 37 |
| Abnormality of the integument | 174 | 27 | 1,171 | 61 | 1,345 | 88 |
| Abnormality of the musculature | 402 | 41 | 1,388 | 98 | 1,790 | 139 |
| Abnormality of the nervous system | 1,570 | 176 | 6,306 | 675 | 7,876 | 851 |
| Abnormality of the respiratory system | 184 | 13 | 687 | 30 | 871 | 43 |
| Abnormality of the skeletal system | 357 | 30 | 3,092 | 283 | 3,449 | 313 |
| Abnormality of the thoracic cavity | 2 | 0 | 1 | 0 | 3 | 0 |
| Abnormality of the voice | 12 | 2 | 43 | 0 | 55 | é |
| Growth abnormality | 72 | 9 | 657 | 38 | 729 | 47 |
| Neoplasm | 54 | 5 | 95 | 12 | 149 | 17 |
| TOTAL | 5,095 | 605 | 25,103 | 2,104 | 30,198 | 2,709 |


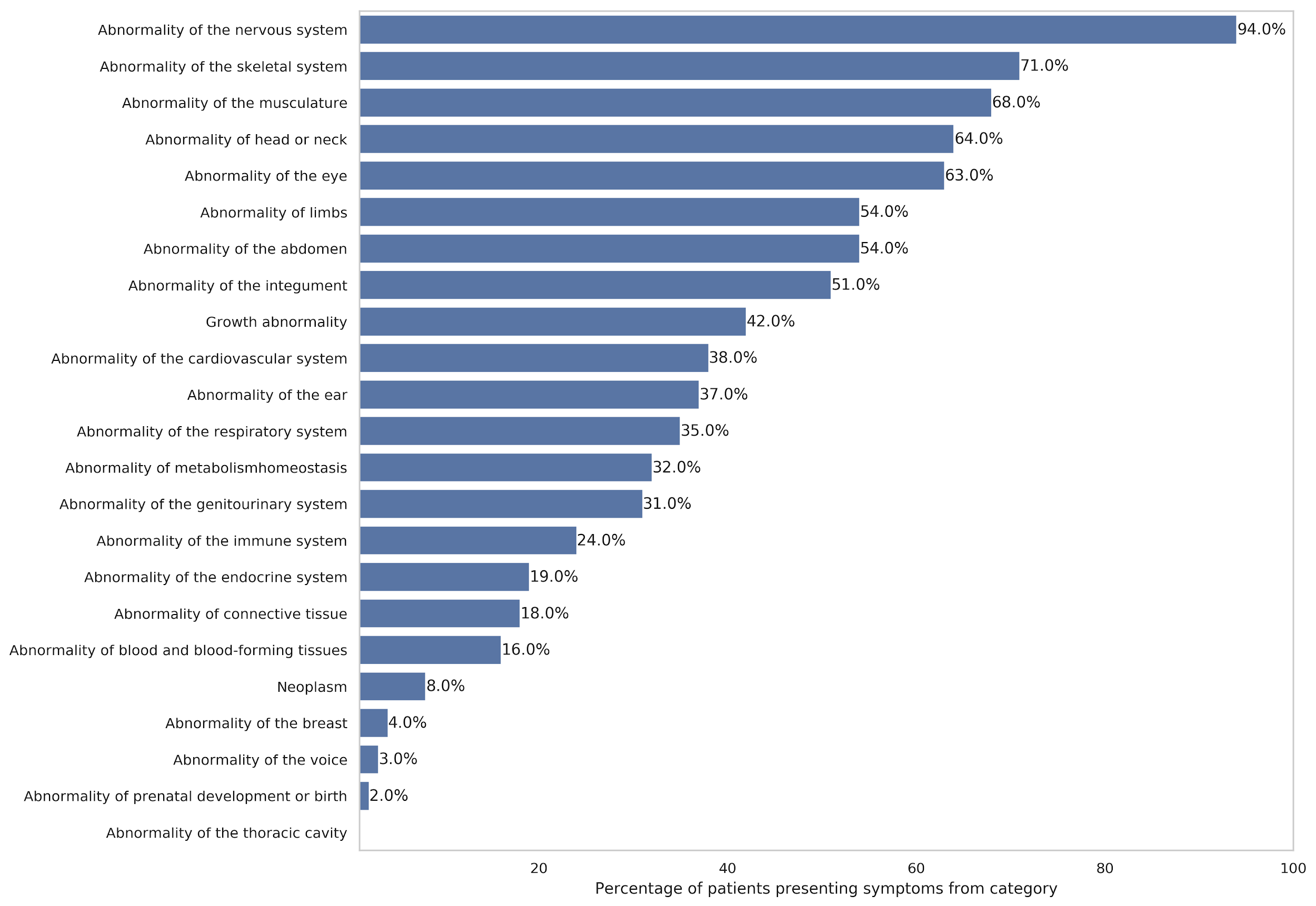


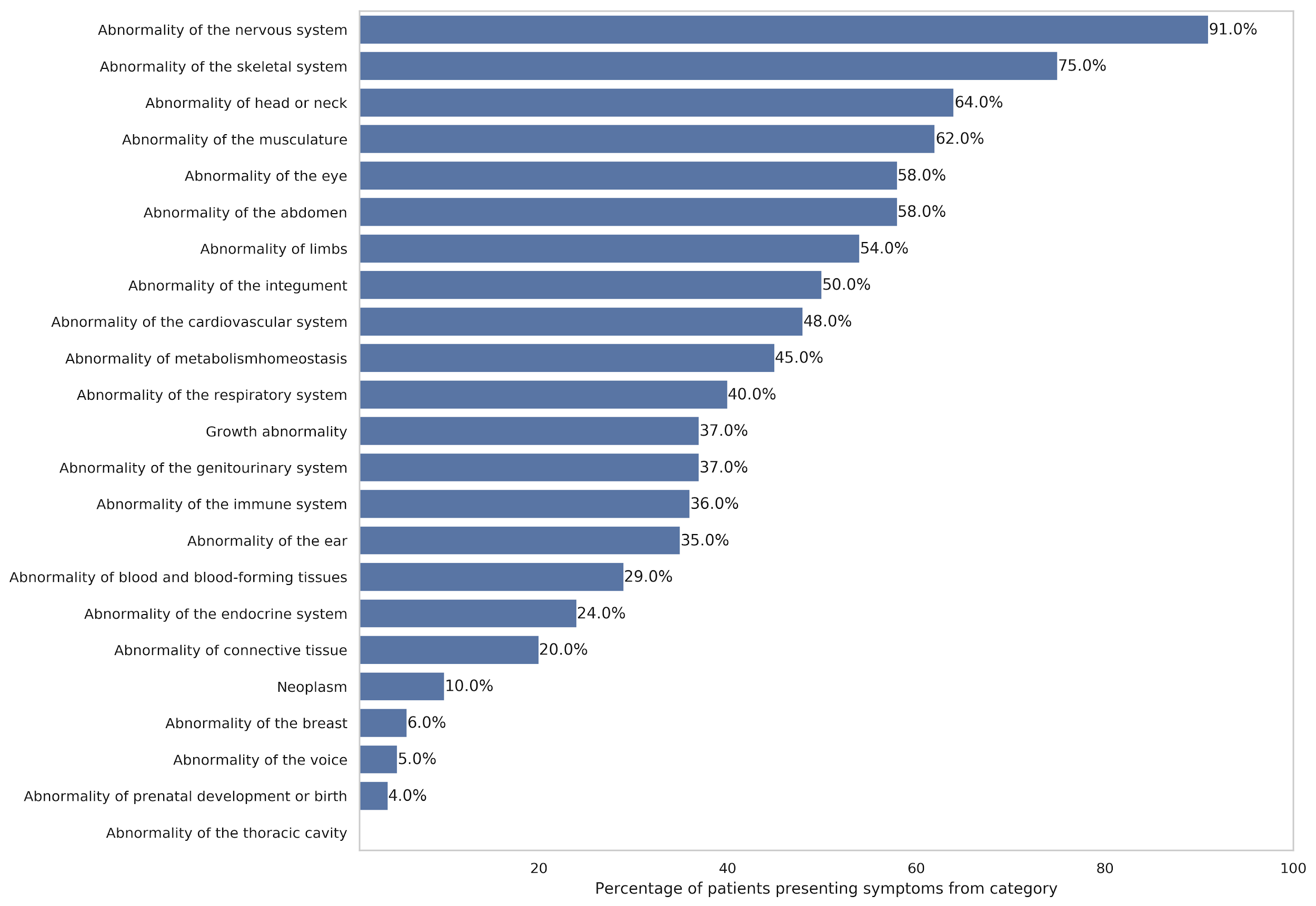


**eFigure 2: Percentage of patients in UDN PIC-SURE database presenting at least one symptom from top-level phenotypic category in HPO, for (a) diagnosed and (b) undiagnosed cases.** There are 23 types of top-level phenotypic abnormalities in which HPO terms can be classified. A single phenotype may be classified within several categories. Each patient was counted within a category if they presented at least one symptom classified in the category (as of May 6th, 2019). The distribution difference was not statistically significant (Mann-Whitney U test: p=0.5, U=264.5).

**Adult network analysis**

The adult network we constructed was formed by 232 nodes (representing patients), with 8,741 edges (representing the similarity between patients). Louvain community detection[^60^](https://paperpile.com/c/RL6PGw/PR0n6) with consensus clustering identified 4 clusters (groups of more than 5 patients) and 8 outliers (groups of less than 5 patients). Outliers were discarded. A total of 220 (95%) adult onset patients were classified within

groups of more than 5 patients.


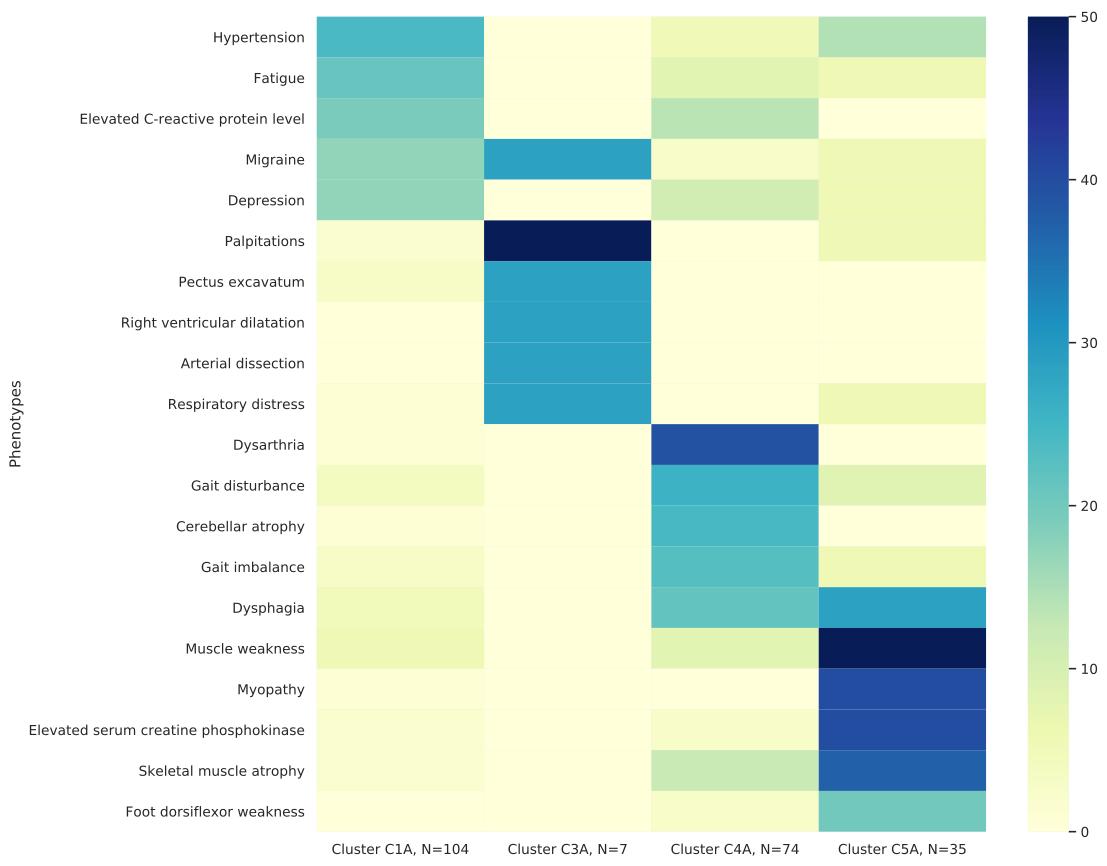


**eFigure 3: Heatmap of most representative phenotypes for each cluster in the UDN for adult network.** The proportion of patients presenting these phenotypes in every cluster is represented in the heatmap, ranging from 0 to 50%.

When grouping adult onset individuals, cluster 1 was characterized by vascular and neuropsychiatric symptoms, specifically hypertension (33%), fatigue (22%), elevated C-reactive protein level (19%), migraine (17%) and depression (17%). Again, this cluster exhibited a significantly increased probability of diagnosis (OR=2.7 CI 95% 1.4 - 5.2). Cluster 2 exhibited cardiological symptoms, including palpitations (57%), pectus excavatum (29%), migraine (29%), right ventricular dilatation (29%) and arterial dissection (29%). Cluster 3 was marked by neuromuscular issues, specifically dysarthria (39%), gait disturbance (26%), cerebellar atrophy (24%), gait imbalance (23%), and dysphagia (22%). Finally, cluster 4 presented characteristics of musculopathies, specifically muscle weakness (54%), myopathy (40%), elevated serum creatine phosphokinase (40%), skeletal muscle atrophy (37%), and dysphagia (29%). Adult clusters only significantly differed in their distribution of HPO terms (p<0.001). There were 31 OMIM[^35^](https://paperpile.com/c/RL6PGw/YEkE)-annotated diagnoses in the network.

Cluster 1 presented a heterogeneous set of diseases such as Alagille (1/1) or Sjörgen’s Syndrome (1/1). Cluster 3 presented neurodegenerative conditions like Alzeihmer (1/1) or Creutzfeldt-Jakob (1/1). Cluster 4 presented myopathies (2/2). Cluster 2 had no associated diagnoses. Spastic Paraplegia and Spinocerebellar Ataxia were found in clusters 1 and 4.

With regard to the adult network, cluster 1 is similar to pediatric cluster 3, presenting symptoms linked to anxiety disorders, as well as adult symptoms such as hypertension[^61^](https://paperpile.com/c/RL6PGw/ml761) and depression.[^62^](https://paperpile.com/c/RL6PGw/DAEPS) Cluster 2 presents cardiovascular phenotypes linked to cardiomyopathies or congenital heart diseases[^63–65^](https://paperpile.com/c/RL6PGw/TmXO+4K7F+5LOX). Cluster 3 is similar to pediatric cluster 4 in neuromuscular symptoms. Finally, cluster 4 presents several phenotypes associated with muscle weakness, linked to myopathies[^66,67^](https://paperpile.com/c/RL6PGw/oUYzz+mwYjg) or motor neuropathies.[^67,68^](https://paperpile.com/c/RL6PGw/WUGYZ+mwYjg)

**eTable 4: Analysis of clusters according to the number of included patients; their female:male ratio; the average number of HPO terms per patient in the cluster; the odds ratio of being diagnosed; the average age at onset of the disease (years) and the average age at UDN evaluation (years) for pediatric patients.** The outliers—clusters with less than 5 patients —were not analyzed. The unspecified or non-applicable values for patients were discarded (as of May 6th, 2019).

|  | Adults | | | | |
| --- | --- | --- | --- | --- | --- |
| Clusters | Cluster C1A | Cluster C2A | Cluster C3A | Cluster C4A | Kruskal-Wallis p-value |
| # of patients per cluster | 104 | 7 | 74 | 35 | / |
| Female:male ratio | 13:10 | 25:10 | 9:10 | 8:10 | / |
| Avg # of HPO terms per patient | 18.3 (95% CI: 15.8 - 20.9) | 9.0 (95% CI: 3.5 - 14.5) | 21.8 (95% CI: 19.4 - 24.1) | 15.0 (95% CI: 12.4 - 17.7) | p<0.001 |
| Odds ratio diagnosed | 2.7 (95% CI: 1.4 - 5.2) | 0.7 (95% CI: 0.1 - 6.2) | 1.1 (95% CI: 0.6 - 2.4) | 1.0 (95% CI: 0.4 - 2.6) | / |
| Average age at onset in y | 38.8 (95% CI: 36.1 - 41.4) | 41.6 (95% CI: 21.7 - 61.4) | 36.7 (95% CI: 33.4 - 40.0) | 38.7 (95% CI: 34.0 - 43.5) | p=0.58 |
| Average age at UDN evaluation in y | 46.7 (95% CI: 42.6 - 50.7) | 47.6 (95% CI: 43.2 - 52.1) | 45.1 (95% CI: 41.8 - 48.4) | 47.8 (95% CI: 44.0 - 51.6) | p=0.73 |

**Validation of cluster comorbidities in national claims database**

**
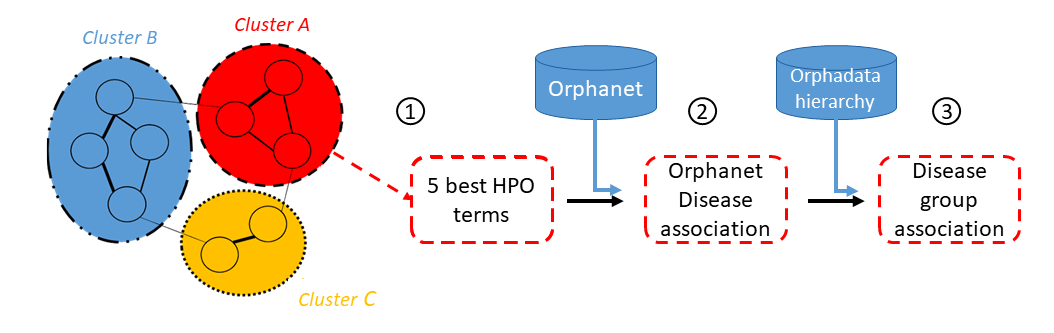
**

**eFigure 4: Workflow for the validation of UDN clustering in the national claims database.** (1) The 5 most represented HPO terms of each cluster were extracted. (2) The HPO terms were translated to Phecode using the approach of Bastarache et al;27 Phecodes were then translated to ICD9/10 codes via the PheWAS catalogue26 to compute the list of associated ICD9/10. (3) Only patients with more than 12 months of coverage and at least one ICD9/10 term mappable to Phecodes were analyzed. For pediatric clusters, only those between 0 and 17 years at first national claims database entry were selected for comparison; for adult clusters, only those more than 18 years old were selected. The claims database patients that present the 5 HPO terms were counted.

We identified 8,697,420 pediatric patients and 30,099,571 adult patients that met the inclusion criteria. We discovered in the claims dataset that the ten comorbidities that were present in the highest proportion of UDN patients in the 3 mappable clusters co-occurred significantly, as compared to non-related comorbidities selected at random, in two of the clusters , (1,000-fold bootstrap evaluated p-value pediatric 2: p=0; pediatric 3: p=0; pediatric 4: p=0.005 ; 500-fold bootstrap evaluated p-value adult 1: p=0.001; adult 2: p>0.05; adult 3: p=0). This statement holds for up to 7, 8 or 9 selected HPO terms out of 10, showing that even a combination of the most representative HPO terms is enriched as compared to random.

**Mapping limitations in national claims database**

Using Bastarache et al[^32^](https://paperpile.com/c/RL6PGw/4KQYc) techniques, we were only able to map 37 of 40 HPO codes associated with pediatric clusters and 38 of 40 HPO codes associated with adult clusters (only 7 out of 10 for pediatric cluster 1 and 8 out of 10 for adult cluster 4). We were thus not able to validate comorbidities associated with pediatric cluster 1 and adult cluster 4 for up to 10 phenotypes.

**References**

60 [Aynaud T. *python-louvain*. Github](http://paperpile.com/b/t4oB6v/G9dKt) <https://github.com/taynaud/python-louvain> [(accessed 12 Apr 2019).](http://paperpile.com/b/t4oB6v/G9dKt)

61 [Pan Y, Cai W, Cheng Q, *et al.* Association between anxiety and hypertension: a systematic review and meta-analysis of epidemiological studies. *Neuropsychiatr Dis Treat* 2015;**11**:1121–30.](http://paperpile.com/b/t4oB6v/kCP6E)

62 [Kendler KS, Neale MC, Kessler RC, *et al.* Major depression and generalized anxiety disorder. Same genes, (partly) different environments? *Arch Gen Psychiatry* 1992;**49**:716–22.](http://paperpile.com/b/t4oB6v/w8yUx)

63 [Pimenta J, Vieira A, Henriques-Coelho T. Ventricular arrhythmia solved by surgical correction of pectus excavatum. *Interact Cardiovasc Thorac Surg* 2018;**26**:706–8.](http://paperpile.com/b/t4oB6v/Qqwzy)

64 [OMIM Entry - # 115200 - CARDIOMYOPATHY, DILATED, 1A; CMD1A.](http://paperpile.com/b/t4oB6v/mgTy7) <https://www.omim.org/entry/115200> [(accessed 30 Oct 2020).](http://paperpile.com/b/t4oB6v/mgTy7)

65 [Abbott AV. Diagnostic approach to palpitations. *Am Fam Physician* 2005;**71**:743–50.](http://paperpile.com/b/t4oB6v/G4K5g)

66 [Chawla J. Stepwise approach to myopathy in systemic disease. *Front Neurol* 2011;**2**:49.](http://paperpile.com/b/t4oB6v/UNWpI)

67 [Yeom J, Song YS, Lee WK, *et al.* Diagnosis and Clinical Course of Unexplained Dysphagia. *Ann Rehabil Med* 2016;**40**:95–101.](http://paperpile.com/b/t4oB6v/mBCMn)

68 [Verschueren A. Motor neuropathies and lower motor neuron syndromes. *Rev Neurol*  2017;**173**:320–5.](http://paperpile.com/b/t4oB6v/G8ijW)

**eTable 5: Associated Diagnoses for patients of the (a) pediatric and (b) adult network.** The diagnoses are annotated using the OMIM[^35^](https://paperpile.com/c/RL6PGw/YEkE) database. The condition as well as the number of patients affected in the cluster are indicated.

**Pediatric network**

| Cluster | Diagnosis, Number of affected patients |
| --- | --- |
| 1 | ACHONDROGENESIS, TYPE IA , 1  AU-KLINE SYNDROME , 1  BAINBRIDGE-ROPERS SYNDROME , 2  BOHRING-OPITZ SYNDROME , 1  COHEN-GIBSON SYNDROME , 1  COMBINED OXIDATIVE PHOSPHORYLATION DEFICIENCY 20 , 1  CONGENITAL DISORDER OF GLYCOSYLATION, TYPE IIm , 1  CONGENITAL HEART DEFECTS, DYSMORPHIC FACIAL FEATURES, AND INTELLECTUAL DEVELOPMENTAL DISORDER , 1  CORNELIA DE LANGE SYNDROME 5 , 1  CYSTIC FIBROSIS , 1  EPILEPSY, PROGRESSIVE MYOCLONIC, 3, WITH OR WITHOUT INTRACELLULAR INCLUSIONS , 1  EPILEPTIC ENCEPHALOPATHY, EARLY INFANTILE, 44 , 1  FLOATING-HARBOR SYNDROME , 1  HYALINE FIBROMATOSIS SYNDROME , 1  HYPOTONIA, INFANTILE, WITH PSYCHOMOTOR RETARDATION AND CHARACTERISTIC FACIES 3 , 2  JOUBERT SYNDROME 30 , 1  LETHAL CONGENITAL CONTRACTURE SYNDROME 7 , 1  MENTAL RETARDATION, AUTOSOMAL DOMINANT 5 , 1  MENTAL RETARDATION, X-LINKED 102 , 1  MENTAL RETARDATION, X-LINKED, SYNDROMIC, TURNER TYPE , 1  MICROCEPHALY 17, PRIMARY, AUTOSOMAL RECESSIVE , 1  MITOCHONDRIAL COMPLEX II DEFICIENCY , 1  MITOCHONDRIAL DNA DEPLETION SYNDROME 6 (HEPATOCEREBRAL TYPE) , 1  NEURODEGENERATION WITH BRAIN IRON ACCUMULATION 3 , 1  NEURODEVELOPMENTAL DISORDER WITH EPILEPSY, CATARACTS, FEEDING DIFFICULTIES, AND DELAYED BRAIN MYELINATION , 1  NEURODEVELOPMENTAL DISORDER WITH HYPOTONIA, SEIZURES, AND ABSENT LANGUAGE , 1  NEURODEVELOPMENTAL DISORDER WITH OR WITHOUT ANOMALIES OF THE BRAIN, EYE, OR HEART , 1  NEURODEVELOPMENTAL DISORDER WITH REGRESSION, ABNORMAL MOVEMENTS, LOSS OF SPEECH, AND SEIZURES , 1  NEURODEVELOPMENTAL DISORDER WITH SPASTIC DIPLEGIA AND VISUAL DEFECTS , 1  OSTEOPATHIA STRIATA WITH CRANIAL SCLEROSIS , 1  PONTOCEREBELLAR HYPOPLASIA, TYPE 2D , 1  SCHAAF-YANG SYNDROME , 2  SHASHI-PENA SYNDROME , 1  SHWACHMAN-DIAMOND SYNDROME 2 , 1  VAN MALDERGEM SYNDROME 2 , 1  WIEDEMANN-STEINER SYNDROME , 1 |
| 2 | 5,10-METHENYLTETRAHYDROFOLATE SYNTHETASE , 1  ALSTROM SYNDROME , 1  AROMATIC L-AMINO ACID DECARBOXYLASE DEFICIENCY , 1  BETHLEM MYOPATHY 1 , 1  CHARCOT-MARIE-TOOTH DISEASE, AXONAL, TYPE 2S , 1  COFFIN-SIRIS SYNDROME 1 , 1  COMBINED OXIDATIVE PHOSPHORYLATION DEFICIENCY 31 , 2  CONGENITAL DISORDER OF GLYCOSYLATION WITH DEFECTIVE FUCOSYLATION 2 , 1  CONGENITAL DISORDER OF GLYCOSYLATION, TYPE IIj , 1  CONGENITAL DISORDER OF GLYCOSYLATION, TYPE Ik , 1  CORTICAL DYSPLASIA, COMPLEX, WITH OTHER BRAIN MALFORMATIONS 3 , 1  DEAFNESS-INFERTILITY SYNDROME , 1  DESANTO-SHINAWI SYNDROME , 1  DYSTONIA, DOPA-RESPONSIVE , 1  EPILEPSY, FOCAL, WITH SPEECH DISORDER AND WITH OR WITHOUT MENTAL RETARDATION , 1  EPILEPSY, HEARING LOSS, AND MENTAL RETARDATION SYNDROME , 1  EPILEPTIC ENCEPHALOPATHY, EARLY INFANTILE, 17 , 1  EPILEPTIC ENCEPHALOPATHY, EARLY INFANTILE, 2 , 1  EPILEPTIC ENCEPHALOPATHY, EARLY INFANTILE, 33 , 1  EPILEPTIC ENCEPHALOPATHY, EARLY INFANTILE, 36 , 1  EPILEPTIC ENCEPHALOPATHY, EARLY INFANTILE, 4 , 1  EPILEPTIC ENCEPHALOPATHY, EARLY INFANTILE, 47 , 1  EPILEPTIC ENCEPHALOPATHY, EARLY INFANTILE, 50 , 1  EPILEPTIC ENCEPHALOPATHY, INFANTILE OR EARLY CHILDHOOD, 2 , 1  EPILEPTIC ENCEPHALOPATHY, INFANTILE OR EARLY CHILDHOOD, 3 , 1  FANCONI ANEMIA, COMPLEMENTATION GROUP R , 1  GASTRIC CANCER, HEREDITARY DIFFUSE , 1  GENERALIZED EPILEPSY WITH FEBRILE SEIZURES PLUS, TYPE 2 , 1  HELSMOORTEL-VAN DER AA SYNDROME , 1  HYPOTONIA, INFANTILE, WITH PSYCHOMOTOR RETARDATION AND CHARACTERISTIC FACIES 2 , 1  HYPOTONIA, INFANTILE, WITH PSYCHOMOTOR RETARDATION AND CHARACTERISTIC FACIES 3 , 1  LEUKODYSTROPHY, HYPOMYELINATING, 6 , 1  MANDIBULOFACIAL DYSOSTOSIS, GUION-ALMEIDA TYPE , 1  MENTAL RETARDATION, AUTOSOMAL DOMINANT 13 , 1  MENTAL RETARDATION, AUTOSOMAL DOMINANT 18 , 2  MENTAL RETARDATION, AUTOSOMAL DOMINANT 20 , 1  MENTAL RETARDATION, AUTOSOMAL DOMINANT 26 , 1  MENTAL RETARDATION, X-LINKED, SYNDROMIC, TURNER TYPE , 1  METABOLIC ENCEPHALOMYOPATHIC CRISES, RECURRENT, WITH RHABDOMYOLYSIS, CARDIAC ARRHYTHMIAS, AND NEURODEGENERATION , 1  MUSCULAR DYSTROPHY, DUCHENNE TYPE , 1  NEUTROPHILIC DERMATOSIS, ACUTE FEBRILE , 1  PITT-HOPKINS-LIKE SYNDROME 2 , 1  PONTOCEREBELLAR HYPOPLASIA, TYPE 6 , 1  RETT SYNDROME , 2  RETT SYNDROME, CONGENITAL VARIANT , 1  ROIFMAN SYNDROME , 1  SPINAL MUSCULAR ATROPHY, TYPE III , 1  STORMORKEN SYNDROME , 1  WIEACKER-WOLFF SYNDROME , 1 |
| 3 | 46,XX SEX REVERSAL 4 , 1  ATTENTION DEFICIT-HYPERACTIVITY DISORDER , 1  AUTOIMMUNE LYMPHOPROLIFERATIVE SYNDROME , 1  BETHLEM MYOPATHY 1 , 1  CILIARY DYSKINESIA, PRIMARY, 7 , 1  CINCA SYNDROME , 1  COFFIN-LOWRY SYNDROME , 1  CONGENITAL HEART DEFECTS, DYSMORPHIC FACIAL FEATURES, AND INTELLECTUAL DEVELOPMENTAL DISORDER , 1  CYSTIC ANGIOMATOSIS OF BONE, DIFFUSE , 1  EHLERS-DANLOS SYNDROME, HYPERMOBILITY TYPE , 1  EPIDERMOLYSIS BULLOSA DYSTROPHICA, AUTOSOMAL RECESSIVE , 1  FACTOR V DEFICIENCY , 1  FAMILIAL COLD AUTOINFLAMMATORY SYNDROME 2 , 1  HASHIMOTO THYROIDITIS , 1  MARFAN SYNDROME , 1  MUSCULAR DYSTROPHY, LIMB-GIRDLE, AUTOSOMAL RECESSIVE 10 , 1  PORETTI-BOLTSHAUSER SYNDROME , 1  PSEUDOPSEUDOHYPOPARATHYROIDISM , 1  SHWACHMAN-DIAMOND SYNDROME 1 , 1  SPINAL MUSCULAR ATROPHY, LOWER EXTREMITY-PREDOMINANT, 1, AUTOSOMAL DOMINANT , 1  STANKIEWICZ-ISIDOR SYNDROME , 1 |
| 4 | ALEXANDER DISEASE , 1  ARTHROGRYPOSIS, DISTAL, WITH IMPAIRED PROPRIOCEPTION AND TOUCH , 1  ATAXIA-TELANGIECTASIA-LIKE DISORDER 1 , 1  BETHLEM MYOPATHY 1 , 1  DYSTONIA 28, CHILDHOOD-ONSET , 1  EPILEPSY, PROGRESSIVE MYOCLONIC 7 , 1  HUNTINGTON DISEASE , 1  HUNTINGTON DISEASE-LIKE 1 , 1  MEGALENCEPHALIC LEUKOENCEPHALOPATHY WITH SUBCORTICAL CYSTS 2B, REMITTING, WITH OR WITHOUT MENTAL RETARDATION , 1  MENTAL RETARDATION, AUTOSOMAL DOMINANT 6, WITH OR WITHOUT SEIZURES , 1  MUCOPOLYSACCHARIDOSIS, TYPE IIIB , 1  MYOPATHY, MYOFIBRILLAR, 1 , 1  NEURODEGENERATION WITH BRAIN IRON ACCUMULATION 2A , 2  NEUROPATHY, HEREDITARY SENSORY, TYPE IF , 1  PORETTI-BOLTSHAUSER SYNDROME , 1  RETT SYNDROME , 1  SPASTIC PARAPLEGIA 11, AUTOSOMAL RECESSIVE , 1  SPASTIC PARAPLEGIA 35, AUTOSOMAL RECESSIVE , 1  SPINOCEREBELLAR ATAXIA, AUTOSOMAL RECESSIVE 8 , 1 |

**Adult network**

| Cluster | Diagnosis, Number of affected patients |
| --- | --- |
| 1 | ALAGILLE SYNDROME 1 1  BASAL GANGLIA CALCIFICATION, IDIOPATHIC, 1 1  CHARCOT-MARIE-TOOTH DISEASE, DEMYELINATING, TYPE 1A 1  CHROMOSOME 1q21.1 DUPLICATION SYNDROME 1  DERMATITIS, ATOPIC, 2 1  EHLERS-DANLOS SYNDROME, CLASSIC TYPE, 2 1  IMMUNODEFICIENCY 21 1  MUSCULAR DYSTROPHY-DYSTROGLYCANOPATHY (LIMB-GIRDLE), TYPE C, 5 1  PARAGANGLIOMAS 1 1  POLYCYSTIC KIDNEY DISEASE 1 WITH OR WITHOUT POLYCYSTIC LIVER DISEASE 1  PSEUDOHYPOPARATHYROIDISM, TYPE IB 1  SJOGREN SYNDROME 1  SPASTIC PARAPLEGIA 9A, AUTOSOMAL DOMINANT 1  SPINOCEREBELLAR ATAXIA 28 1  STIFF-PERSON SYNDROME 1  WILLIAMS-BEUREN SYNDROME 1 |
| 2 | NONE |
| 3 | ADRENOLEUKODYSTROPHY 1  ALZHEIMER DISEASE 3 1  BASAL GANGLIA CALCIFICATION, IDIOPATHIC, 1 1  BROWN-VIALETTO-VAN LAERE SYNDROME 1 1  CREUTZFELDT-JAKOB DISEASE 1  FRONTOTEMPORAL DEMENTIA ANDOR AMYOTROPHIC LATERAL SCLEROSIS 1 1  MUCOPOLYSACCHARIDOSIS, TYPE IIIC 1  MUSCULAR DYSTROPHY, CONGENITAL, MEGACONIAL TYPE 1  PEROXISOME BIOGENESIS DISORDER 14B 1  SPASTIC PARAPLEGIA 7, AUTOSOMAL RECESSIVE 1  SPINOCEREBELLAR ATAXIA 8 1  SPONGIFORM ENCEPHALOPATHY WITH NEUROPSYCHIATRIC FEATURES 1 |
| 4 | GLYCOGEN STORAGE DISEASE XV 1  MYOPATHY, DISTAL, 5 1  MYOPATHY, MYOFIBRILLAR, 8 1 |

**Members of the Undiagnosed Diseases Network**

Maria T. Acosta

Margaret Adam

David R. Adams

Pankaj B. Agrawal

Mercedes E. Alejandro

Patrick Allard

Justin Alvey

Laura Amendola

Ashley Andrews

Euan A. Ashley

Mahshid S. Azamian

Carlos A. Bacino

Guney Bademci

Eva Baker

Ashok Balasubramanyam

Dustin Baldridge

Jim Bale

Michael Bamshad

Deborah Barbouth

Gabriel F. Batzli

Pinar Bayrak-Toydemir

Anita Beck

Alan H. Beggs

Gill Bejerano

Hugo J. Bellen

Jimmy Bennet

Beverly Berg-Rood

Raphael Bernier

Jonathan A. Bernstein

Gerard T. Berry

Anna Bican

Stephanie Bivona

Elizabeth Blue

John Bohnsack

Carsten Bonnenmann

Devon Bonner

Lorenzo Botto

Lauren C. Briere

Elly Brokamp

Elizabeth A. Burke

Lindsay C. Burrage

Manish J. Butte

Peter Byers

John Carey

Olveen Carrasquillo

Ta Chen Peter Chang

Sirisak Chanprasert

Hsiao-Tuan Chao

Gary D. Clark

Terra R. Coakley

Laurel A. Cobban

Joy D. Cogan

F. Sessions Cole

Heather A. Colley

Cynthia M. Cooper

Heidi Cope

William J. Craigen

Michael Cunningham

Precilla D'Souza

Hongzheng Dai

Surendra Dasari

Mariska Davids

Jyoti G. Dayal

Esteban C. Dell'Angelica

Shweta U. Dhar

Katrina Dipple

Daniel Doherty

Naghmeh Dorrani

Emilie D. Douine

David D. Draper

Laura Duncan

Dawn Earl

David J. Eckstein

Lisa T. Emrick

Christine M. Eng

Cecilia Esteves

Tyra Estwick

Liliana Fernandez

Carlos Ferreira

Elizabeth L. Fieg

Paul G. Fisher

Brent L. Fogel

Irman Forghani

Laure Fresard

William A. Gahl

Ian Glass

Rena A. Godfrey

Katie Golden-Grant

Alica M. Goldman

David B. Goldstein

Alana Grajewski

Catherine A. Groden

Andrea L. Gropman

Sihoun Hahn

Rizwan Hamid

Neil A. Hanchard

Nichole Hayes

Frances High

Anne Hing

Fuki M. Hisama

Ingrid A. Holm

Jason Hom

Martha Horike-Pyne

Alden Huang

Yong Huang

Rosario Isasi

Fariha Jamal

Gail P. Jarvik

Jeffrey Jarvik

Suman Jayadev

Yong-hui Jiang

Jean M. Johnston

Lefkothea Karaviti

Emily G. Kelley

Dana Kiley

Isaac S. Kohane

Jennefer N. Kohler

Deborah Krakow

Donna M. Krasnewich

Susan Korrick

Mary Koziura

Joel B. Krier

Seema R. Lalani

Byron Lam

Christina Lam

Brendan C. Lanpher

Ian R. Lanza

C. Christopher Lau

Kimberly LeBlanc

Brendan H. Lee

Hane Lee

Roy Levitt

Richard A. Lewis

Sharyn A. Lincoln

Pengfei Liu

Xue Zhong Liu

Nicola Longo

Sandra K. Loo

Joseph Loscalzo

Richard L. Maas

Ellen F. Macnamara

Calum A. MacRae

Valerie V. Maduro

Marta M. Majcherska

May Christine V. Malicdan

Laura A. Mamounas

Teri A. Manolio

Rong Mao

Kenneth Maravilla

Thomas C. Markello

Ronit Marom

Gabor Marth

Beth A. Martin

Martin G. Martin

Julian A. Martínez-Agosto

Shruti Marwaha

Jacob McCauley

Allyn McConkie-Rosell

Colleen E. McCormack

Alexa T. McCray

Heather Mefford

J. Lawrence Merritt

Matthew Might

Ghayda Mirzaa

Eva Morava-Kozicz

Paolo M. Moretti

Marie Morimoto

John J. Mulvihill

David R. Murdock

Avi Nath

Stan F. Nelson

John H. Newman

Sarah K. Nicholas

Deborah Nickerson

Donna Novacic

Devin Oglesbee

James P. Orengo

Laura Pace

Stephen Pak

J. Carl Pallais

Christina GS. Palmer

Jeanette C. Papp

Neil H. Parker

John A. Phillips III

Jennifer E. Posey

John H. Postlethwait

Lorraine Potocki

Barbara N. Pusey

Aaron Quinlan

Wendy Raskind

Archana N. Raja

Genecee Renteria

Chloe M. Reuter

Lynette Rives

Amy K. Robertson

Lance H. Rodan

Jill A. Rosenfeld

Robb K. Rowley

Maura Ruzhnikov

Ralph Sacco

Jacinda B. Sampson

Susan L. Samson

Mario Saporta

C. Ron Scott

Judy Schaechter

Timothy Schedl

Kelly Schoch

Daryl A. Scott

Lisa Shakachite

Prashant Sharma

Vandana Shashi

Jimann Shin

Rebecca Signer

Catherine H. Sillari

Edwin K. Silverman

Janet S. Sinsheimer

Kathy Sisco

Kevin S. Smith

Lilianna Solnica-Krezel

Rebecca C. Spillmann

Joan M. Stoler

Nicholas Stong

Jennifer A. Sullivan

Angela Sun

Shirley Sutton

David A. Sweetser

Virginia Sybert

Holly K. Tabor

Cecelia P. Tamburro

Queenie K.-G. Tan

Mustafa Tekin

Fred Telischi

Willa Thorson

Cynthia J. Tifft

Camilo Toro

Alyssa A. Tran

Tiina K. Urv

Matt Velinder

Dave Viskochil

Tiphanie P. Vogel

Colleen E. Wahl

Stephanie Wallace

Nicole M. Walley

Chris A. Walsh

Melissa Walker

Jennifer Wambach

Jijun Wan

Lee-kai Wang

Michael F. Wangler

Patricia A. Ward

Daniel Wegner

Mark Wener

Monte Westerfield

Matthew T. Wheeler

Anastasia L. Wise

Lynne A. Wolfe

Jeremy D. Woods

Shinya Yamamoto

John Yang

Amanda J. Yoon

Guoyun Yu

Diane B. Zastrow

Chunli Zhao

Stephan Zuchner
